# The role of an intermediate unit in a clinical pathway

**DOI:** 10.5334/ijic.859

**Published:** 2013-03-26

**Authors:** Anne-Kari Johannessen, Hilde Lurås, Sissel Steihaug

**Affiliations:** HØKH/Research Senter, Akershus University Hospital, 1478 Lørenskog, Norway; HØKH/Research Senter, Akershus University Hospital, 1478 Lørenskog, Norway; HØKH/Research Senter, Akershus University Hospital, 1478 Lørenskog, Norway, SINTEF Technology and Society, Health Research, Box 124 Blindern, N-0314 Norway

**Keywords:** intermediate unit, clinical pathways, older, qualitative research

## Abstract

**Introduction:**

Different care models have been established to achieve more coordinated clinical pathways for older patients in the transition between hospital and home. This study explores an intermediate unit’s role in a clinical pathway for older patients with somatic diseases.

**Theory and methods:**

Qualitative data were collected via interviews, observations, and a questionnaire. Participants included patients and healthcare providers within both specialist and primary healthcare. Transcripts of interviews and field notes were analyzed using a method of systematic text condensation.

**Results:**

Healthcare providers in the hospital, the intermediate unit, and the municipalities have different opinions about who is a ‘suitable’ patient for the unit and what is the proper time for hospital discharge. This results in time-consuming negotiations between the hospital and the unit. Incompatible computer systems increase the healthcare provider’s workload. Several informants are doubtful as to whether a stay in the unit is useful to the patients, while the patients are mostly pleased with their stay and the transferral.

**Conclusion and discussion:**

This study describes challenges that may occur when a new unit is established in an existing healthcare system in order to achieve an appropriate clinical pathway from hospital to home.

## Introduction

Demographic changes in the Western world are resulting in an increased number of older people with chronic diseases. This implies a need for better organized and more efficient healthcare services, including the better use of resources in the hospital sector [1, 2]. The Norwegian healthcare system is divided into two separate governmental levels: the specialist and the primary healthcare system. Norwegian hospitals are organized within the specialist healthcare system, while the municipalities hold the responsibility for primary healthcare. The two levels act in accordance with different laws, regulations, goals, and tasks. A constant challenge is to improve the coordination between the two levels. The Coordination Reform white paper—Report no. 47 to the Storting “Proper treatment—at the right place and right time”—is one initiative aimed at meeting the demands for better coordination and more efficient use of resources in the Norwegian healthcare system [3]. The reform was officially implemented on January 1, 2012. To improve coordination one measure is to develop a pathway described as a chronologic chain of events that makes up the patient’s meeting points with different areas of healthcare services [3]. The literature uses various concepts to describe the patient’s pathway through the healthcare system. Clinical pathway is frequently used [4, 5], and for this paper we have chosen this term. Clinical pathways are used throughout the world in different kinds of healthcare settings [6], and are viewed as multidisciplinary tools that can be used to organize the care processes in order to improve quality and efficiency [6, 7]. The term can also be defined as a method for the patient-care management of well-defined groups of patients during a well-defined period of time [4]. The pathway that is investigated in this research paper focuses on older patients with somatic diseases but is not restricted to any certain diagnosis. A clinical pathway can be used as a tool to achieve a continuum of care across settings [4, 6], and intermediate care (IC) is a service often used to achieve this continuum.

Several definitions of IC are in use, and the term IC hints at care as something ‘in between’ [8–10]. IC can be understood as services or activities concerned with a patient’s transition from the hospital to home, and from medical/social dependence to functional independence [9]. The main issues associated with IC are as follows: prevent unnecessary hospital admission and prolonged hospital stay, facilitate timely hospital discharge, compose structured individual care plans, and maximise independent living. The services are performed by cross-professional work and limited to six weeks [10–12].

Several qualitative studies have revealed some similarities associated with IC when it becomes a part of the traditional healthcare services for older people regardless of the organisational frame for the IC services (e.g. from acute hospitals, community rehabilitation beds, day hospital, rapid response team, Hospital at Home, etc.) [10, 13–16]. IC is seemingly associated with increased quality of life and enables patients to be more independent. Furthermore, IC is perceived as more flexible and as having more resources to offer for the recovery of patients [10, 14]. A more ‘home-like environment’ and less institutional features of IC facilities are considered important contributors to a good recovery process [14, 16]. Lack of awareness and comprehension about the content and meaning of IC is reported as a weakness. Indeed, the concept is seemingly not deeply rooted within health professions [10, 14]. Another feature is insufficient involvement of physicians [13, 14]. A national pilot audit of IC conducted in the UK showed that the majority of admissions to IC services are considered either as a ‘step-down’ from acute hospital wards or as a ‘step-up’ from the patient’s home. The most common reasons for admission to IC include rehabilitation after medical illness such as pneumonia and falls, with or without fracture [12].

Over the last few decades, the establishment of IC units has been one of several initiatives introduced to develop better clinical pathways for older patients [17–20]. IC units aim to bridge the gap between healthcare services and healthcare providers in order to improve coordination and integration [13, 21, 22]. Nurse-led units (NLU) and community hospitals are two different types of IC units [22]. An NLU can be described as an institutional setting where nurses are primarily responsible for care management, including admission and discharge decisions, and team leadership. A distinct admission criterion into an NLU is that patients must be deemed medically stable but not ready for discharge when they are transferred to an NLU [23]. Some evidence indicates that patients discharged from an NLU are better prepared for discharge, but patients also tend to have longer inpatient stays in such units [24]. Increased functional status may simply be a product of the increased length of inpatient stay, but this is not clear. No statistically significant adverse effects were noted, but the possibility of increased early mortality in an NLU cannot be discounted (ibid.). A study that focused on the economic evaluation of an NLU as compared to standard care in an acute ward showed that both inpatient costs and total costs were significantly higher for the NLU [25].

A community hospital can be described as a small hospital that does not offer diagnostic facilities or specialised services [16, 17]. In his doctoral thesis, Garåsen found that treatment and care in a community hospital resulted in reduced mortality, fewer re-hospitalizations, and greater cost-effectiveness as compared to treatment in a general hospital department [18]. Another study of community hospitals shows significantly greater functional independence at six months for patients allocated to one of seven community hospitals [17]. In a complementary cost-effectiveness study, O’Reilly et al. found that the health outcomes and costs between the two services were similar [26].

Even with several studies investigating the costs and usefulness of IC units based on quantitative and qualitative research designs, there is still limited knowledge about an IC unit in collaboration with different healthcare institutions and levels of healthcare.

In recent years a considerable number of intermediate units have been established in Norway. These include units with different organizing, staffing, patients groups and capacity. Some units are organized within the hospitals others are governed by the municipalities, and yet others are collaborations between the specialist and the primary healthcare system. Most units are designed to take care of patients in the pathway from hospital to home, and some are dedicated to prevent hospital admission. In addition to nurses, most intermediate units are staffed by physicians, physiotherapists, and occupational therapists. Older patients with orthopaedic diagnosis or heart and lung conditions are the most common patients groups admitted to the units. Average length of stay at the different units is between two and three weeks. The size of the units varies from five to thirty beds.

## Objective

The objective of this study is to explore an intermediate unit’s role in the clinical pathway from hospital to home for older patients with somatic diseases.

## Methods

### Material

The intermediate unit studied has several similarities with an NLU and is a collaboration between a University Hospital and four municipalities in the hospital catchment area. There are 460,000 inhabitants belonging to the hospital’s catchment area, of whom 120,000 live in the four municipalities involved in this collaboration. The purpose of the unit is to contribute to improving the clinical pathway for patients aged 60 and over with somatic diseases who are in transition from hospital to home. The aim is that all patients should be discharged to their own home and to manage at home with or without municipal home-based assistance after a stay at the unit. The intermediate unit opened in 2009 and has fifteen beds. The signed contract of agreement states that the unit shall offer medical treatment, rehabilitation, and nursing. The maximum length of stay in the unit is three weeks. The municipalities are responsible for the registered and enrolled nurses and for the non-medical staff (the posts of secretary and janitor), while the hospital contributes a physician, an occupational therapist, and a physiotherapist, all of whom hold 50% positions. A nurse leads the unit and is also in charge of the admission and discharge decisions of patients. Healthcare providers and leaders from the hospital, the unit, and the municipalities had agreed on the admission criteria prior to the opening of the unit.

Table 1 shows the form used in the hospital’s admission to the unit.

In 2009, 2010 and 2011 respectively 254, 294 and 269 patients stayed in the unit. Nearly half of the patients were diagnosed with orthopaedic conditions, often caused by hip surgery. Other groups were patients diagnosed with lung and heart conditions, patients suffering from infections, and patients with cancer.

The qualitative data are drawn from interviews, observation, and a short questionnaire. Combining several methods provides access to different contexts and gives a more comprehensive picture of the situation.

### Interviews

Qualitative interviews are suitable when the intention is to explore personal experiences and the meaning people associate with these [27]. Forty-six persons, including patients and healthcare providers, were interviewed by the first author. The interviews were semi-structured, based on Kvale’s principles [28] and were carried out from March 2009 until June 2011. The interviews lasted about one hour, were all recorded on digital recording equipment, and were transcribed nearly verbatim by the interviewer.

Eight patients—four women and four men, 63 to 83 years old—were recruited by the first author with assistance of the nursing staff in the unit. They were treated for hip fracture, infection, or pain after cancer surgery. The patients were interviewed twice: first during their stay in the unit, and then two or three weeks after discharge to their own home. The patients were asked about their stay in the unit, the transition from the hospital to the unit and from the unit to their own home, and how they experienced the first weeks at home.

Healthcare providers within the unit and the municipalities were recruited by the first author in cooperation with the head nurse of the unit. For these informants we applied a purposeful sampling strategy covering healthcare providers with different backgrounds regarding age, work experience, professional background, and current position in the unit or in the municipalities (Table 2).

The average age of all healthcare providers was 42. Seven of the municipal informants worked as leaders in the long-term care division, five worked in the office that processed requests for municipality services (orderer office), and two held clinical positions. Fifteen of the unit informants held clinical positions, while one worked as a leader. The recruitment of hospital informants was done by ‘snowball sampling’ in the hospital departments that transfer most patients to the unit [29]. Three of the hospital informants were leaders, five held clinical posts, and one held a coordinator position. The providers were interviewed at their workplace and were asked about their work and their collaboration with each other.

### Observations

Observations were performed at six multidisciplinary meetings, six report meetings, and four assessment meetings during the same period that the interviews were conducted. Observation is an appropriate method for understanding actions and interactions that people themselves are not aware of [27, 30]. The purpose of the multidisciplinary meetings was to make up plans for the patient’s further stay in the unit. Members of the different professions working in the unit attended these meetings. In the report meetings registered and enrolled nurses discussed nursing tasks that should be performed the same day. Patients, their next of kin, and healthcare providers from the unit and the municipality attended the assessment meetings where the patient’s needs for municipality care after discharge were discussed.

During the observations the researcher had a passive, non-participant role, and immediately after the meetings she wrote down her observations as clearly and as accurately as possible (observation notes). Additionally, she wrote down her reflections after every observation (reflective notes). The reflection and observation notes constitute the field notes in the study.

### Questionnaire

Physicians have unique positions in hospitals, however, no hospital physicians responded positively to the request to be interviewed. A short questionnaire was therefore sent out to a random sample of 25 hospital physicians working in the same departments as the hospital nurse informants. The questionnaire included a few qualitative questions on the physician’s experiences with the intermediate unit and had open fields intended for free text.

A total of 14 physicians, eight men and six women, responded. Five physicians stated that they were familiar with the unit while nine had less knowledge about it.

## Analysis

Transcripts of interviews, field notes, and text from questionnaires constitute the qualitative data in this study. The data were analysed by systematic text condensation as described by Malterud [31]. Two of the authors (the nurse and the physician) participated in the analysing process. We conducted the analysis in four steps, alternating between the various steps throughout the entire process. The first analytic step involved reading all the material to obtain an overall impression. In the second step we identified themes representing different aspects of the patients’ and providers’ experiences of the services and work in the unit, and coded these under different headings—for instance ‘suitable patients’ or “challenging application procedure”. In the third analytic step we condensed and abstracted the contents of each coded group. In the fourth step, we summarized the knowledge of each coded group to provide a generalized description that reflected the main findings of the patients’ and the employees’ experiences, and compared the results with the interview statements to make sure that our concepts were grounded in the data. We then selected one or more quotations from the data material that appropriately illustrated the meaning of the descriptions. Finally we developed the headers that summarized the content of each coded group.

## Ethics

Written, informed consent was obtained from the healthcare providers and the patients. The study was approved by the local privacy legislation authority at the University Hospital. The application was submitted to the Regional Committee for Medical and Health Research. The project was not found to be part of the Committee’s mandate since it is not regarded as medical or healthcare research conducted with the purpose of generating knowledge about illness or health.

The intermediate unit and the municipal long-term care services were in the first place unknown fields to the authors. However, the hospital involved is the first author’s former workplace, and although she knew some of the informants by name she had never worked directly with any of them.

## Results

### Disagreement and negotiations about ‘suitable’ patients

Although the hospital, the unit, and the municipalities have a mutual signed contract about the admission criteria to the unit, discussion and disagreement about the criteria and about ‘suitable’ patients for a stay in the unit were main issues in the interviews. Providers in the unit thought the criteria were clear and easy to deal with, while most hospital providers perceived the criteria as rigorous and wanted a more flexible practice. Several hospital physicians wrote in the questionnaire that they found the criteria too narrow and argued that more patients could have profited from a stay in the unit. The discussions on the criteria and ‘suitable’ patients were described as a mixture of negotiations, demands, and expectations. Most informants from the municipalities experienced the criteria as straightforward but a little restricted: for instance they asserted that patients with more extended needs for rehabilitation and patients with less serious dementia would have profited from a stay. There were often disagreements between the collaborative partners about whether patients were to be characterized as mentally ill or suffering from dementia. One provider from the municipalities said:

“Many of our patients have multiple diagnoses and can often be depressed, so if we are to follow the agreed criteria absolutely there are quite a few who are included to have a stay in the unit”.

The physiotherapist and the occupational therapist working in the unit wanted more patients with a need for interdisciplinary approaches in order to utilize their own competence. The unit’s employees emphasized that medical treatment should not be finished when patients were transferred to the unit, while the hospital employees wanted to keep the patients until their treatment was completed. More than half of the hospital informants stated that the notion “completed medical treatment” was discussed at length between providers working in the hospital and in the unit. They emphasized that patients can have a great need for physical training and supportive treatment—for example supportive oxygen therapy—even after the medical treatment is officially finished. A nurse in the hospital expressed it as follows:

“We had a very suitable patient for the unit, but she was regarded as having completed treatment and so there was no place for her in the unit. There are written rules about how to define a patient who is ready to be discharged, but they don’t necessarily work in practice”.

Employees in the unit claimed that the hospital often transferred patients either too early or too late during their period of treatment, and argued that this made their tasks more challenging. A nurse and a physiotherapist working in the unit were of the view that some patients had an unnecessary stay in the unit and instead could have managed with a couple more days in the hospital before they were discharged directly to their home. They questioned if the unit stay only prolonged the total time spent in institutions. Hospital employees were also preoccupied with this situation. One said:

“We have to be really careful here because sometimes we transfer patients to the unit far too early. But I also think that patients become even more ill if they stay in hospital longer than absolutely necessary”.

Hospital providers found it challenging to select the correct patients for the unit. They might have found a ‘suitable’ patient, informed the patient, prepared all the documents, and then recognised that the patient’s resident municipality was not one of the four included in the collaboration.

According to informants in the unit, they periodically received patients who completely infringed the criteria: they suffered from dementia or they had an extended need for care because they had several diseases. Observations in the unit showed that ‘suitable’/‘not suitable’ patients were a recurrent issue at the healthcare providers’ meetings. Some providers at the unit asserted that the unit would constantly have vacant beds if they followed the admission criteria strictly, and observations also showed that the unit periodically had several vacancies.

### Challenging application procedure and transfer to the unit

According to employees working in the unit, extensive information has been provided to the hospital about the unit, the admission criteria, and the application procedure. Nevertheless these subjects result in considerable frustration among employees in both the hospital and the unit. In the initial phase, patients’ applications to the unit were made by calling the unit, but from autumn 2010 the hospital staff had to use an electronic form. Although the new procedure simplified the unit’s work situation, the hospital employees experienced this routine as an extra workload and were worried about whether an electronic form could replace valuable dialogue with the unit. One hospital nurse said:

“The unit expects so many forms and documents from us. They wait for medication lists, medical records, nurses’ reports and a clear rehabilitation plan. There’s an awful lot of work to be done, and all the same it’s not sure that the patient will be given a place”.

Five out of eight patients said they were poorly prepared for the transfer to the unit. They could not remember having received any information about the unit or being asked whether they wanted a stay there. Several patients were indignant about the fact that they were obliged to pay. One patient said:

“I’m astonished that I have to make a personal contribution. Why should the treatment I was given free in hospital suddenly cost me money”?

Hospital informants found the long waiting time for replies and the repeated refusals demotivating for new applications. Many informants, independent of workplace, wanted special hospital coordinators who could assist in the application process and patient selection.

The hospital and the unit had different computer systems, which meant that the hospital had to fax the printed form to the unit. Incompatible computer systems also resulted in extra work when the patients were discharged from the unit to the municipality. Considerable time was spent repeating information and double-checking information that stemmed from telephone calls, faxes, and letters. However, all the patients emphasized that the unit seemed to have good and updated knowledge about them.

### Resources and being busy

All the healthcare providers experienced being in a situation with scarce resources, but it was a general consensus that the hospital was the most vulnerable. Several informants from the municipalities and the hospital claimed that the unit was privileged because it had stable resources, i.e. a minimal turn-over of staff, as well as the opportunity to control who was to be admitted to the unit, and it was easy to acquire a clear overview of the activities. Some hospital physicians wished that the unit had the ability to increase patient admissions in busy periods. Hospital nurses said their staffing was too low to allow them to take care of the patients in the manner they wanted to. One hospital head nurse described the hospital pressure in the following way:

“In the hospital we can only take one day at a time. We just can’t manage to think about the future or in the long-term. This means that in the hospital the job of getting older people on their feet and walking around can’t be given priority”.

The patients also commented on the bustle in the hospital. One of them said:

“It was terribly hectic in the hospital, an almost frightening tempo! I didn’t like being there—they just rushed past me and there wasn’t much help available”.

Employees in the unit said that time pressure and lack of resources forced the hospital into a practice they characterized as ‘throwing patients out’. They reported that applications to the unit were low in periods when the hospital was under extra pressure. Municipal employees asserted that being busy and under constant pressure were customary in primary health care and were rarely debated anymore. They asserted that they had little time for patient care, and physical training was not on their list of tasks. One nurse in the home-based services said:

“The clock is my worst enemy. I’ve been given more and more to do, and anyway I don’t have time to help the patients with exercise. So they fall back into the old habits when they come home. In addition the health of our patients is steadily getting worse”.

Informants from three orderer offices said that they encouraged the hospital to send patients to the unit in order to relieve the municipal services for a while. They also reported that the hospital staff caused extra work for the municipalities because they quite often simultaneously applied for patient beds in ordinary municipal nursing homes and in the intermediate unit, i.e. the hospital simultaneously activated two administrative systems. On the other hand, several informants from the municipalities experienced that the pressure on traditional nursing homes was reduced after the intermediate unit opened.

### Different opinions about the unit and its tasks

Most informants from the municipalities and the hospital were of the view that the unit offered good healthcare and emphasized the combined medical and rehabilitation service provided. They pointed to the calm surroundings, good food, and pleasant social atmosphere as important qualities of the unit. Several hospital physicians were doubtful about the patients’ benefit of a stay in the unit. Nurses representing the home-based services claimed that they did not notice any difference between patients discharged from the unit and other patients with regard to physical activity and mastering everyday life. Several patients were pleased with the service and their stay in the unit. One patient stated:

“This is a wonderful service, and much better than what they promise in their brochure”.

Other patients found the rooms dreary and felt that the rooms complicated normal activity. In addition, some patients felt unsafe because there was no ‘24-hour physician’ present. Although six of the eight patients had more than a three-week stay in the unit, they still experienced the first weeks at home as difficult, and most of them were dependent on their next of kin. Observations showed that on several occasions patients, their next of kin and healthcare providers agreed to extend the stay in the unit. Three patients described it this way:

“It was depressing to come home after such a great stay in the short-term unit”. “I didn’t want to go home but I felt that I had no choice”.

“I feel I get too little help from the home-based care services. They should be here at least once a day”.

The providers had different opinions about what kinds of tasks this unit should perform. The hospital and municipal informants asserted that the unit’s main task was helping patients exercise and assisting them in their rehabilitation and that the employees should pay less attention to medical treatment. The unit informants on the other hand expressed that both activities were equally important. The unit’s employees characterized the unit as “a place to regain strength”, “a medical treatment institution” or “a rehabilitation institution”. Several of the unit employees felt they neither belonged to a hospital nor a municipal system: they found themselves in between the two levels because the daily tasks, procedures, and the need for knowledge were different from those of their colleagues in the hospital and the municipalities.

## Discussion

### ‘Suitable’ patients—‘suitable’ for whom?

The results show that the employees in the hospital and the intermediate unit spend considerable time and effort negotiating about who is a ‘suitable’ patient and what is a proper time for transferring patients to the unit. Hospital work is performed in accordance with the Norwegian legislation relating to the special healthcare service, and the main task is to carry out medical treatment [32]. When the course of treatment is completed, the hospital has fulfilled its tasks, and the patient should be discharged as soon as possible. However, patients who in the opinion of the hospital are ready to be discharged are not necessarily ‘suitable’ patients for the unit. ‘Suitable’ patients for the unit are those who recover sufficiently and manage at home after a stay of three weeks. The unit offers medical treatment, rehabilitation, and nursing, and has competence that enables them to take care of further treatment and follow-up. It follows that the unit prefers patients that have not completed their treatment and who are in need of rehabilitation and interdisciplinary services. This is in accordance with other studies describing the struggle of finding ‘suitable’ patients for an intermediate unit [10, 13].

Several informants from the hospital and the municipalities describe the unit’s position as protected and privileged. The results indicate that the unit can define patient groups and tasks more easily than the hospital and hence, has a more predictable situation as well as more time to take care of the patients than is the case in the hospital. Considering the hospital’s and the unit’s different situations and commitments, it is reasonable that negotiations and disagreements take place.

According to the Norwegian legislation relating to municipal health and care services, the municipalities are obliged to take care of all patients discharged from the hospital and the unit [33]. The home-based services are responsible for stabilizing, rehabilitation, and nursing, and a ‘holistic’ and long-term perspective is necessary. The municipalities have scarce resources, and the employees describe how they struggle to get patients admitted to the unit and keep them there as long as possible in order to reduce the pressure on their own services. Christensen et al. describe how different organizational goals can express conflicting interests and can result in tensions between cooperative participants [34]. The three cooperative partners have different commitments, goals, and tasks, and the results of our study illustrate how conflicting goals create tension and challenge the cooperation.

### The role of the unit in an appropriate clinical pathway

An important aim of initiatives like this intermediate unit is to achieve improved clinical pathways. The results show that the informants have different opinions as to whether a stay in the unit is useful to the patients. Overall, informants in the study experience the unit as an excellent service to patients. At the same time many of the informants, and mainly those representing the hospital, are doubtful about the unit’s usefulness to patients and that it only extends the total institutional stay for the patients. The patients feel safe and are given good care and assistance in the unit. Many patients, however, needed an extended stay and several patients experienced the transition to their home as difficult. Increased length of stay seems to be the case in nurse led intermediate units compared to treatment in general hospital departments [21, 22, 24].

Regardless of employment, the healthcare providers perceive the unit as an ‘in-between’ unit [8]. This suggests a more independent position, and the unit is likely to be considered as a new administrative level. This may imply unnecessary bureaucratization. Non-communicating computer systems enhance the impression of an ‘in-between unit’.

Despite much effort and preparation, such as repeated information about the unit and considerable work to put new procedures and routines in place, the results show that transferring patients generates challenging and time-consuming negotiations and a great deal of work for the healthcare providers. Time-consuming assessment procedures are in accordance with results from other studies [13]. We find that applications decrease in particularly busy periods for the hospital. This indicates that the referral procedure is too extensive, and that the hospital’s healthcare providers are better off when patients are discharged to the municipality according to the ordinary well-established discharge routines rather than following this new arrangement.

The results show that the hospital informants experience that many of their applications to the unit are rejected. The intermediate unit has 15 beds and 460,000 inhabitants live in the hospital’s catchment area. Only four of the 24 municipalities in the hospital’s catchment area are entitled to dispose the unit’s beds. This indicates that the unit’s capacity hardly fulfils hospital needs. It is then reasonable to ask whether a unit dedicated to a small number of patients belonging to a limited number of municipalities is worth the effort of finding ‘suitable’ patients to transfer.

Different forms of communication and different understandings of services, as well as lack of knowledge and insight into the other provider’s roles and obligations, are frequent obstructions to teamwork [35]. A changed application procedure from phone call to electronic application form involves less verbal contact between the hospital and the unit, and is an example of a barrier between teams.

Distinctly different clinical roles, responsibilities, and approaches can hamper coordinated and integrated clinical pathways [36]. The providers in this study hold different tasks and goals and do not necessarily have a common understanding of the patients’ needs. However, they may have a common understanding, but their collaboration is restrained by conditions such as low staffing, huge workload, and incompatible computer systems. It is easier to understand the other party’s rationality when both parties know each other. Building up a network and strengthening human relations presuppose time, good will, and meeting points.

### Discussion of methods

In this study we chose a combination of interviews, observation, and questionnaires. We argue that this gave us broader data and a more secure basis for analysis and interpretation than one data source alone. The interviews provided knowledge that was useful for observing in meetings. Observation gave a better insight into practical conditions that patients recognized as crucial for a good transition to home. Informants from different occupational groups provided varied and multifaceted information. Combining data from both providers and patients gave a more complete picture of the services and the experiences of transitions.

The first author conducted and transcribed all the interviews. By virtue of her background as a nurse she had expertise in the field. This made it easy for her to understand what happened in the unit, but also led her to disregard or not notice episodes that were taken for granted for a nurse. The latter was counteracted through discussing the findings from the interviews and observations with her supervisors and by her own reflections on these issues. The first author and the main supervisor (the physician) conducted some of the analysis together. The results were also discussed with research colleagues. Preliminary results were presented to the unit’s employees, and they recognized their own situations in the descriptions and were conversant with the interpretations of the results.

## Conclusion

This study describes challenges that may occur when a new unit is established in an existing healthcare system. The results demonstrate how the different goals and tasks of the three institutions challenge and hamper the collaboration. If an intermediate unit is to become an integrated part of an appropriate clinical pathway in a complex healthcare system, considerable effort in establishing procedures and routines for transferring patients is required. The results indicate that it is challenging for the collaborating partners to develop joint overarching objectives and see their own services as part of a wider perspective.

## Reviewers

**Marie-Aline Bloch**, Dean of Research and of Innovative Teaching & Learning, EHESP School of Public Health, France

**Etsuji Okamoto**, Prof. Dr., National Institute of Public Health, Department of Management Sciences, Japan

One anonymous reviewer.

## Figures and Tables

**Table 1.**
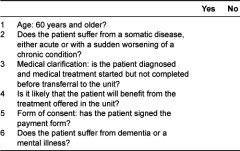
Admission criteria

**Table 2.**
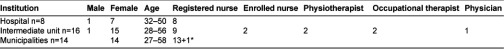
Healthcare providers interviewed

*One leader in the long-term care division had a background as a social educator, the others were registered nurses.

## References

[1] Helse- og omsorgsdepartementet (2005). Fra stykkevis til helt. En sammenhengende helsetjeneste.

[2] European Commission (2008). Long-term care in the European Union.

[3] Helse- og omsorgsdepartementet (2009). Samhandlingsreformen. Rett behandling-på rett sted-til rett tid.

[4] De Bleser L, Depreitere R, De Waele K, Vanhaecht K, Vlayen J, Sermeus W (2006). Defining pathways. Journal of Nursing Management.

[5] Kinsman L, Rotter T, James E, Snow P, Willis J (2010). What is a clinical pathway? Development of a definition to inform the debate. BioMedCentral Medicine.

[6] Vanhaecht KF, De Witte KF, Panella MF, Sermeus W (2009). Do pathways lead to better organized care processes?. Journal of Evaluation in Clinical Practice.

[7] Vanhaecht K, Bollmann M, Bower K, Gallagher C, Gardini A, Guezo J (2006). Prevalence and use of clinical pathways in 23 countries—an international survey by the European Pathway Association. Journal of Integrated Care Pathways.

[8] Melis RJ, Olde Rikkert M, Olde Rikkert MG, Parker S, Parker SG, van Eijken M (2004). What is intermediate care?. British Medical Journal.

[9] Steiner A (2001). Intermediate care—a good thing?. Age and Ageing.

[10] Glasby J, Martin G, Regen E (2008). Older people and the relationship between hospital services and intermediate care: results from a national evaluation. Journal of Interprofessional Care.

[11] Young J (2009). The development of intermediate care services in England. Arcives of Gerontology and Geriatrics.

[12] Hutchinson T, Young J, Forsyth D (2011). National pilot audit of intermediate care. Clinical Medicine.

[13] Plochg T, Delnoij DM, van der Kruk TF, Janmaat TA, Klazinga NS (2005). Intermediate care: for better or worse? Process evaluation of an intermediate care model between a university hospital and a residential home. BioMedCentral Health Services Research.

[14] Regen E, Martin G, Glasby J, Hewitt G, Nancarrow S, Parker H (2008). Challenges, benefits and weaknesses of intermediate care: results from five UK case study sites. Health & Social Care in the Community.

[15] Wiles RF, Postle KF, Steiner AF, Walsh B (2003). Nurse-led intermediate care: patients’ perceptions. International Journal of Nursing Studies.

[16] Green J, Forster A, Young J, Small N, Spink J (2008). Older people’s care experience in community and general hospitals: a comparative study. Nursing Older People.

[17] Young J, Green J, Forster A, Small N, Lowson K, Bogle S (2007). Postacute care for older people in community hospitals: a multicenter randomized, controlled trial. Journal of the American Geriatrics Society.

[18] Garåsen H (2008). The Trondheim model: improving the professional communication between the various levels of health care services and implementation of intermediate care at a community hospital could provide better care for older patients: short and long-term effects. PhD Thesis.

[19] Helse- og omsorgsdepartementet (2011). Helsetjenester til syke eldre.

[20] Romøren TI, Torjesen DO, Landmark B (2011). Promoting coordination in Norwegian health care. International Journal of Integrated Care.

[21] Steiner A (2001). Intermediate care: more than ‘a nursing thing’. Age and Ageing.

[22] Griffiths PF, Edwards MF, Forbes AF, Harris R (2005). Post-acute intermediate care in nursing-led units: a systematic review of effectiveness. International Journal of Nursing Studies.

[23] Steiner AF, Walsh BF, Pickering RM, Wiles RF, Ward JF, Brooking JI (2001). Therapeutic nursing or unblocking beds? A randomised controlled trial of a post-acute intermediate care unit. British Medical Journal.

[24] Griffiths PD, Edwards MH, Forbes A, Harris RL, Ritchie G (2007). Effectiveness of intermediate care in nursing-led in-patient units. Cochrane Database Syst Review.

[25] Walsh B, Steiner A, Pickering RM, Ward-Basu J (2005). Economic evaluation of nurse led intermediate care versus standard care for post-acute medical patients: cost minimisation analysis of data from a randomised controlled trial. British Medical Journal.

[26] O’Reilly J, Lowson K, Green J, Young J, Forster A (2008). Post-acute care for older people in community hospitals—a cost-effectiveness analysis within a multi-centre randomised controlled trial. Age and Ageing.

[27] Malterud K (2001). The art and science of clinical knowledge: evidence beyond measures and numbers. The Lancet.

[28] Kvale S, Brinkmann S (2009). Interviews: learning the craft of qualitative research interviewing.

[29] Heckathorn DD (2002). Respondent-driven sampling II: deriving valid population estimates from chain-referral samples of hidden populations. Social Problems.

[30] Mays N, Pope C (1995). Qualitative research: observational methods in health care settings. British Medical Journal.

[31] Malterud K (1993). Shared understanding of the qualitative research process. Guidelines for the medical researcher. Family Practice.

[32] Helse- og omsorgsdepartementet (2011). Lov 1999-07-02 nr 61 om spesialisthelsetjenesten. Ikrafttredelse 1973-03-20, endret 2011-12-16.

[33] Helse- og omsorgsdepartementet (2012). Lov om kommunale helse- og omsorgstjenester.

[34] Christensen T, Lægreid P, Roness PG, Rørvik KA (2007). Organization theory and the public sector: instrument, culture and myth.

[35] Maslin-Prothero SE, Bennion AE (2010). Integrated team working: a literature review. International Journal of Integrated Care [serial online].

[36] Kodner DL, Spreeuwenberg C (2002). Integrated care: meaning, logic, applications, and implications—a discussion paper. International Journal of Integrated Care [serial online].

